# Concentration of non-myocyte proteins in arterial media of cerebral autosomal dominant arteriopathy with subcortical infarcts and leukoencephalopathy

**DOI:** 10.1371/journal.pone.0281094

**Published:** 2023-02-08

**Authors:** Soo Jung Lee, Akhil Kondepudi, Kelly Z. Young, Xiaojie Zhang, Naw May Pearl Cartee, Jijun Chen, Krystal Yujin Jang, Gang Xu, Jimo Borjigin, Michael M. Wang

**Affiliations:** 1 Department of Neurology, University of Michigan, Ann Arbor, MI, United States of America; 2 Neurology Service, VA Ann Arbor Healthcare System, Department of Veterans Affairs, Ann Arbor, MI, United States of America; 3 Molecular and Integrative Physiology, University of Michigan, Ann Arbor, MI, United States of America; Zhejiang University School of Medicine, CHINA

## Abstract

The most common inherited cause of vascular dementia and stroke, cerebral autosomal dominant arteriopathy with subcortical infarcts and leukoencephalopathy (CADASIL), is caused by mutations in NOTCH3. Post-translationally altered NOTCH3 accumulates in the vascular media of CADASIL arteries in areas of the vessels that exhibit profound cellular degeneration. The identification of molecules that concentrate in the same location as pathological NOTCH3 may shed light on processes that drive cytopathology in CADASIL. We performed a two phase immunohistochemical screen of markers identified in the Human Protein Atlas to identify new proteins that accumulate in the vascular media in a pattern similar to pathological NOTCH3. In phase one, none of 16 smooth muscle cell (SMC) localized antigens exhibited NOTCH3-like patterns of expression; however, several exhibited disease-dependent patterns of expression, with antibodies directed against FAM124A, GZMM, MTFR1, and ST6GAL demonstrating higher expression in controls than CADASIL. In contrast, in phase two of the study that included 56 non-SMC markers, two proteins, CD63 and CTSH, localized to the same regions as pathological NOTCH3, which was verified by VesSeg, a customized algorithm that assigns relative location of antigens within the layers of the vessel. Proximity ligation assays support complex formation between NOTCH3 fragments and CD63 in degenerating CADASIL media. Interestingly, in normal mouse brain, the two novel CADASIL markers, CD63 and CTSH, are expressed in non-SMC vascular cells. The identification of new proteins that concentrate in CADASIL vascular media demonstrates the utility of querying publicly available protein databases in specific neurological diseases and uncovers unexpected, non-SMC origins of pathological antigens in small vessel disease.

## Introduction

Small vessel disease of the brain plays an important role as a cause of vascular dementia and stroke as a cofactor in the progression of Alzheimer’s disease and related dementias [1–4]. The most common inherited cause of small vessel disease is the monogenic disorder, cerebral autosomal dominant arteriopathy with subcortical infarcts and leukoencephalopathy (CADASIL) [5, 6]. CADASIL is caused by mutations in NOTCH3 that result in a change in the number of cysteine residues in the gene product [7, 8]. While the initiating genetic events in CADASIL have been determined, the ensuing molecular changes in blood vessels that ultimately result in vascular failure have not been completely delineated.

The initial site of pathology in CADASIL is presumed to be the arterial media which is composed of vascular smooth muscle cells (SMC), the principal site of NOTCH3 gene expression and protein deposition [9–13]. Disease-related post-translational modification of NOTCH3 occurs most prominently in the medial layer of cerebral arteries in this disease. For example, conformations of NOTCH3 that correspond to multiple reduced cysteine forms of the protein have been shown to deposit in the media [14]. Truncation of the protein between the first and second EGF-like repeats occurs primarily in the media [10]. Further, oligomeric complexes of NOTCH3 fragments, visualized by proximity ligation assays, reside in media [15]. Recently, we identified a second cleavage event in NOTCH3, which also occurs predominantly in vascular media [11]. These modified forms of NOTCH3 are enriched in CADASIL and concentrate in the vascular media of both small and large vessels.

In addition to NOTCH3, protein components of CADASIL arterial media include type IV collagen [16], VTN [17], BGN [18], DCN [19], TIMP3 [17], as well as other proteins [20, 21]. The accumulation of these proteins occurs in a characteristic pattern in arteries, with much high levels in the media relative to the intima. Identification of additional molecules co-habiting within the vascular media with NOTCH3 forms could shed light on both adaptive factors and active participants in disease pathways [17].

In prior work, we tested whether or not canonical smooth muscle marker proteins accumulate in the media with NOTCH3 in CADASIL vessels. Surprisingly, each of the five canonical smooth muscle cell marker proteins studied exhibited redistribution in vessels with a deficiency of these proteins in the media and buildup within the intima of brain arteries [22]. Here, we performed an extended immunohistological screen to identify proteins that localize with NOTCH3 in the pathological media of CADASIL.

Candidates for the current extended screen include probes that we have curated from a large scale visual screen of the Human Protein Atlas [23] which identified a series of novel proteins that are expressed selectively in vessels (versus neuroglia) within the human brain [24, 25]. We initially focused on the expression of 16 non-canonical SMC markers in CADASIL brain with the goal of uncovering resident vascular medial proteins that accumulate within the smooth muscle layer of diseased brain. None of the non-canonical SMC markers accumulated in the degenerating media of CADASIL vessels; however, we subsequently uncovered two non-SMC vascular proteins that concentrate in media of CADASIL. In light of these results, we discuss the cellular origins of molecules that concentrate in the vascular media of CADASIL.

## Methods

### Immunohistochemistry

Formalin fixed frontal lobes sections that have been previously described [11] or obtained from the Alzheimer’s Disease Center at the University of Michigan and the Brain Bank of the National Institute for Developmental and Childhood Disorders at the University of Maryland were used. At least 8 samples were stained for each antibody; the average age of death for controls was 68 years and for CADASIL was 66 years. Paraffin embedded sections (five microns in thickness) were mounted on plus slides and analyzed using chromogenic immunohistochemical staining using antibodies after citrate-induced antigen retrieval [10, 11]. Staining was followed by hematoxylin counterstaining. Human brain vessel protein integrity was assessed using the monoclonal antibody BRIC231 (anti-H; Santa Cruz).

Immunohistochemistry was performed using antibodies purchased from Sigma: ADIRF (Sigma cat# HPA026810), AGXT (Sigma cat# HPA035371), DES (Sigma cat# HPA018803), EPX (Sigma cat# HPA050507), FAM124A (Sigma cat# HPA048182), GPX8 (Sigma cat# HPA036720), GZMM (Sigma cat# HPA015624), LRRC41 (Sigma cat# HPA051941), MTFR1 (Sigma cat# HPA023152), NEURL4 (Sigma cat# HPA055314), PRMT2 (Sigma cat# HPA018976), ST6GALNAC6 (Sigma cat# HPA018890), TBC1D2B (Sigma cat# HPA052663), TEX261 (Sigma cat# HPA016631), CD63 (Santa Cruz cat# sc-15363 or DHSB cat# H5C6), and CTSH (Sigma cat# HPA003524). The antibodies used in the Human Protein Atlas for LPHN2/ADGRL2 showed weak staining in mouse tissues, and the product was discontinued; therefore, independent reagents for ADGRL2 were employed to confirm results (LSBio cat# LS-B7725-50).

### Quantitative RT-PCR

RNA was extracted and purified from frozen brain tissue using an RNeasy kit (Qiagen). Subsequently, RNA was reverse transcribed to cDNA by standard methods using random primers, and cDNA was quantified by real-time PCR. All experiments were carried out in triplicate. A list of primer sequences used can be found in Table 1. Expression levels shown are normalized to 18S ribosomal RNA whose concentrations were determined in parallel qPCR reactions.

**Table 1:** Primer sequences against human cDNA sequences used for qRT-PCR. Sequences are listed in the 5’ to 3’ orientation.

Gene names	Forward	Reverse
**Human 18S**	**CAGCCACCCGAGATTGAGCA**	**TAGTAGCGACGGGCGGTGTG**
**ADGRL2**	**CTGGAGACGGGATATGAAGAT**	**GGATAATTCTCGCCTCACCA**
**ADIRF**	**GCAGAAAGCCATGGACCA**	**CAGACCGAGGAGGTGGTG**
**AGXT**	**GCCCAGGATGTACCATCACAC**	**GTCATAGCCAGCGGGTACAG**
**AOC3**	**TGCTGGAAAGGATTTGGTG**	**GGACCGCCTAGTTGTGAGAG**
**CTSH**	**GGATGTCTAAGCACCGTAAGAC**	**GCAATTCTGAGGCTCTGACC**
**CD63**	**TCCATAAGGAGGGCTGTGT**	**TCCCAAAACCTCGACAAAAG**
**DES**	**GCATGAAGAGGAGATCCGTG**	**AGAAATGTTCTTAGCCGCGA**
**EPX**	**CCCAGAAGAGCATCAAGCAG**	**CAGTGATGGTGCGGTACTTG**
**FAM124A**	**CTCAGGTGAGCTTTCCGTT**	**CTCTTCGCCGTACTCCTCCT**
**GPX8**	**TGGAAAAGTATAAAGGCAAAGTTTC**	**AAAAGCCAACACGCTGAAGT**
**GZMM**	**CTGCTTCAGCTGGACGGGAA**	**GTGTCCAGCACTTGGAGGTC**
**LRRC41**	**CCTCAAGAAAGGCCTCTCAAC**	**CACAAAGACGCCTGTCAGAA**
**MTFR1**	**CATGCAATCGGTACTTTGGTC**	**CTTCTTTGGCTACCCATCCA**
**NEURL4**	**CGCATAATGACTTTGCCAAC**	**GCGCTCAGCCGTCTTATTAT**
**PRMT2**	**TGAATCGCAGGGAGAAGAGC**	**GAGTTCCATAGCTGCCGAAG**
**ST6GAL**	**TAGCAACAAAGAGCAGCGGT**	**GACAATCACACACTGGTGGC**
**TBC1D2B**	**GTGCTCAAGGCTCCCAATC**	**AGCATTTGGGTGTGGGTAAA**
**TEX261**	**CTGTCGTGTGGACTAGTGGTG**	**ATCTCCTGGCTGCATGGTAG**

### Proximity ligation assay

Five-micron formalin-fixed frontal lobe sections were prepared for PLA using standard immunohistochemical methods, including citrate-mediated antigen retrieval. The primary antibodies for CD63 (H5C6) and NOTCH3 fragmentation product (UMI-F) were detected using oligonucleotide-labeled anti-mouse and anti-rabbit secondary antibodies, respectively. PLA was performed according to manufacturer’s brightfield PLA instructions (Sigma-Aldrich), except for increasing the amplification time to 5 hours [15].

### In situ hybridization

The location of mRNA corresponding to CD63 and CTSH in CADASIL frontal lobe was assessed using a system designed by Advanced Cell Diagnostics. The protocol, provided by the manufacturer, involved hybridization of custom designed nucleic acid probes that were detected using multiple non-enzymatic amplification steps followed by chromogenic detection using an alkaline phosphatase-conjugated terminal probe as previously described [15, 18]. Signal was documented using brightfield microscopy for magenta puncta.

### Data analysis

Staining shown is representative across at least two independent rounds of immunohistochemistry. Scoring of tissue staining was performed by investigators blinded to diagnosis. We report only human/mouse IHC differences which were independently and consistently noted by all investigators. Vessels that showed staining with acceptable signal to noise free from artefacts were selected for image analysis by the customized algorithm noted below. Quantitative RT-PCR analysis was performed using the Mann-Whitney U-test with significance considered as p<0.05.

### Image segmentation

Image analysis was accomplished by sequential application of ImageJ and a custom algorithm written for this study called VesSeg. This workflow consists of manual and automatic processing stages. The manual steps involve removing the image background, leaving the vessel wall. Then, the stained regions within the vessel wall are isolated by employing a thresholding criterion. Concurrently, the vessel wall is circumferentially segmented into 100 areas of equal width. Then, the number of stained pixels within each area is determined, resulting in a staining distribution of the vessel. Staining distributions for vessels stained for the same marker are then averaged and compared to other markers. The specifics of the algorithm are described in the S1 File, and definition of terms used for image segmentation are displayed in S1 Fig in S1 File.

### Quantitation and component analysis of staining

Multiple representations of staining patterns in the blood vessel walls were produced for analysis. We assumed the tunica intima, media, and adventitia were all equal width in all vessel walls. Similarity between protein staining distributions was calculated as the inverse of their Jensen-Shannon distance. The square root of the Jensen-Shannon divergence (JSD) is a commonly used method to measure the similarity between two probability density functions [26].

Next, to score each arterial layer across vessels stained for a marker, the mean staining distribution, consisting of 100 bins, were divided into 3 groups (e.g., 0 to 33, exclusive for the tunica intima) whose values were then summed, yielding three values. To determine statistical significance between the layers, an unpaired, one-sided, two-sample t-test was performed between the following pairs: (intima, media) and (media, adventitia). The permutation test described by Hernandez-Morera and colleagues was also used to determine the statistical significance of differences in staining between arterial layers [27].

Finally, we utilized t-distributed stochastic neighbor embedding (t-SNE) to visualize the staining patterns for each marker. Specifically, this was performed by dividing the vessel into 20 tangential segments of equal width, calculating staining distributions for each patch, and then applying t-SNE. The resulting analyses were plotted and compared between markers. The code for VesSeg and subsequent quantification performed can be found at https://github.com/michaelwanglab/vesseg.

## Results

### Expression of 16 non-canonical SMC markers in vascular layers

**Fig 1:**
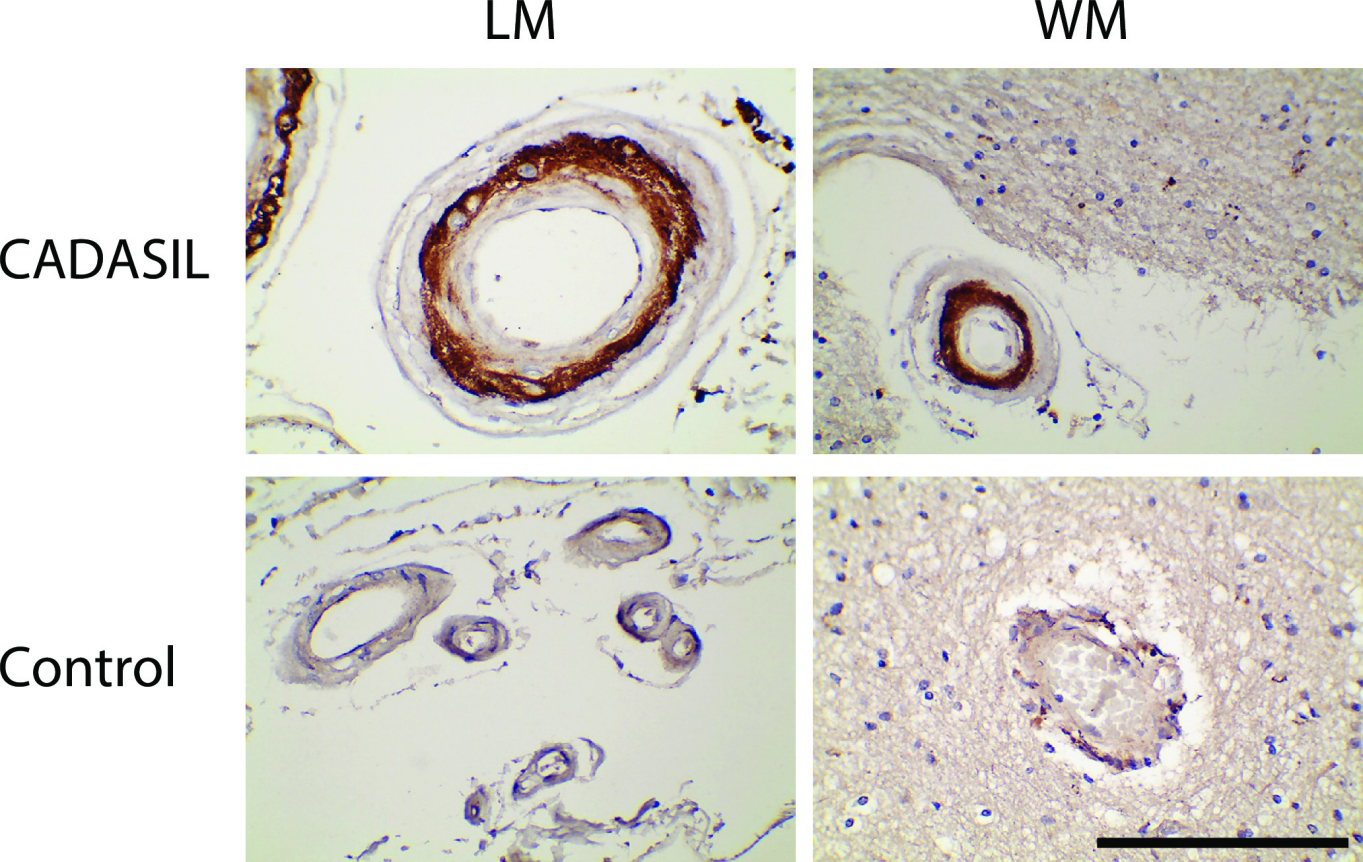
NOTCH3 protein localization in CADASIL and control arteries. Localization of NOTCH3 fragmentation products was performed in CADASIL (top row) and non-CADASIL frontal cortex by immunohistochemistry with UMI-F, which recognizes the neo-epitope generated by fragmentation of NOTCH3 at the junction between EGF-like repeats 1 and 2. The left panels show leptomeningeal arteries (LM) and the right panels show white matter arteries (WM). Arteries from both regions demonstrate medial staining corresponding to the vascular smooth muscle layer of arteries with deficiency in the intimal and adventitial layers. Scale bar shows 100 microns.

**Fig 2:**
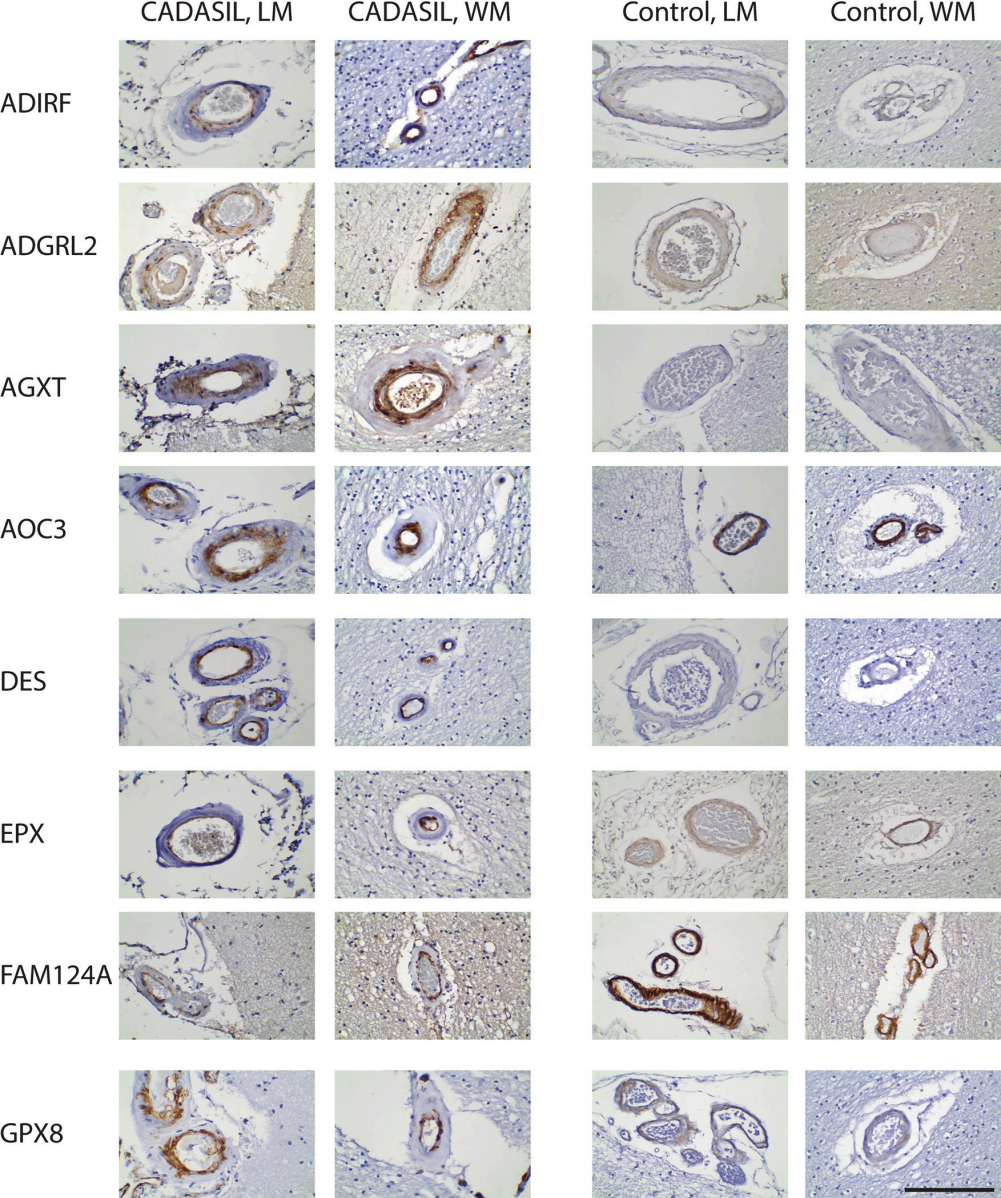
Vascular smooth muscle cell proteins in CADASIL and control arteries. Eight antibodies (labeled on the left) were used for immunohistochemical localization of respective smooth muscle antigens in CADASIL and control brains. Both leptomeningeal (LM) and white matter (WM) vessels are shown to illustrate representative positively-stained vessels. Scale bar shows 100 microns.

**Fig 3:**
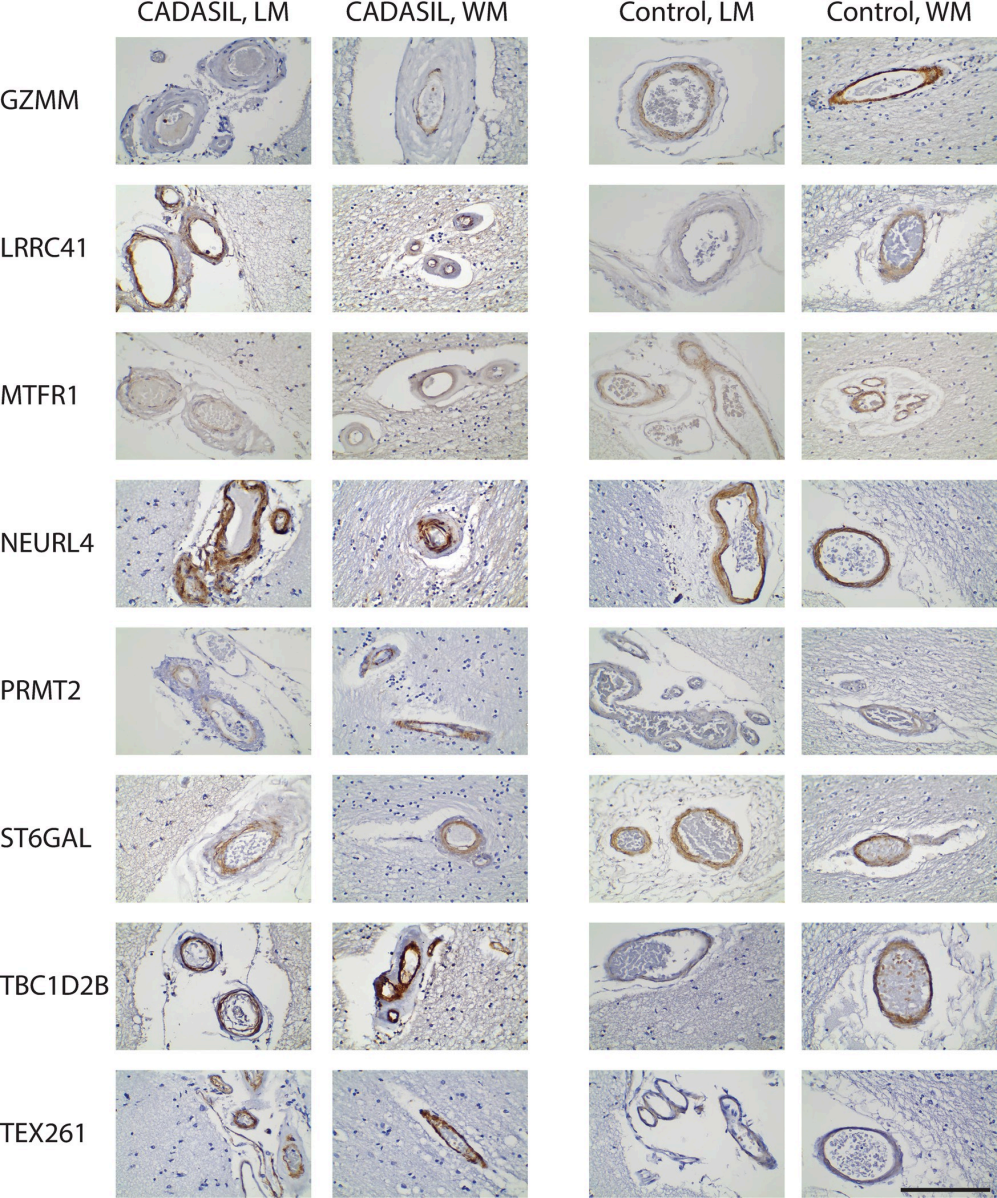
Vascular smooth muscle cell proteins in CADASIL and control arteries. Eight antibodies (labeled on the left) were used for immunohistochemical localization of respective smooth muscle antigens in CADASIL and control brains. Both leptomeningeal (LM) and white matter (WM) vessels are shown to illustrate representative positively-stained vessels. Scale bar shows 100 microns.

We initially hypothesized that a selected group of SMC proteins accumulate in CADASIL vascular media, the site of NOTCH3 accumulation (Fig 1). To test this, we selected probes that have been shown to react reliably against archival brain tissue and which specifically highlight SMC. In a previous study, we characterized 16 non-canonical SMC molecules in human brain arterial SMC proteins and showed that they were largely conserved in SMC of vascular and non-vascular tissue and across multiple species, with uncommon exceptions [24]. These proteins were independent from canonical SMC markers which were previously examined [22]. Antibody probes to these 16 non-canonical SMC molecules were applied to frontal lobe sections from genetically defined CADASIL patients to investigate their pattern of expression in diseased tissue (Figs 2 and 3). We assessed whether any of the 16 markers met two criteria that are characteristic features of NOTCH3 ectodomain and NOTCH3 cleavage product distribution in CADASIL: 1) media greater than intima and adventitia expression and 2) increased expression in CADASIL media over control media [10–12, 14].

We observed differences between the 16 markers in their zonal expression within arteries (Figs 2 and 3), but we did not find that any of the markers localized in a fashion similar to NOTCH3; specifically, none of the 16 markers exhibited medial layer concentration in degenerating smooth muscle that has been seen with NOTCH3 expression in CADASIL. A common pattern of expression was concentration of staining in the intima of CADASIL vessels (most notable in ADIRF, DES, EPX, FAM124A, GZMM, and TEX261).

**Fig 4:**
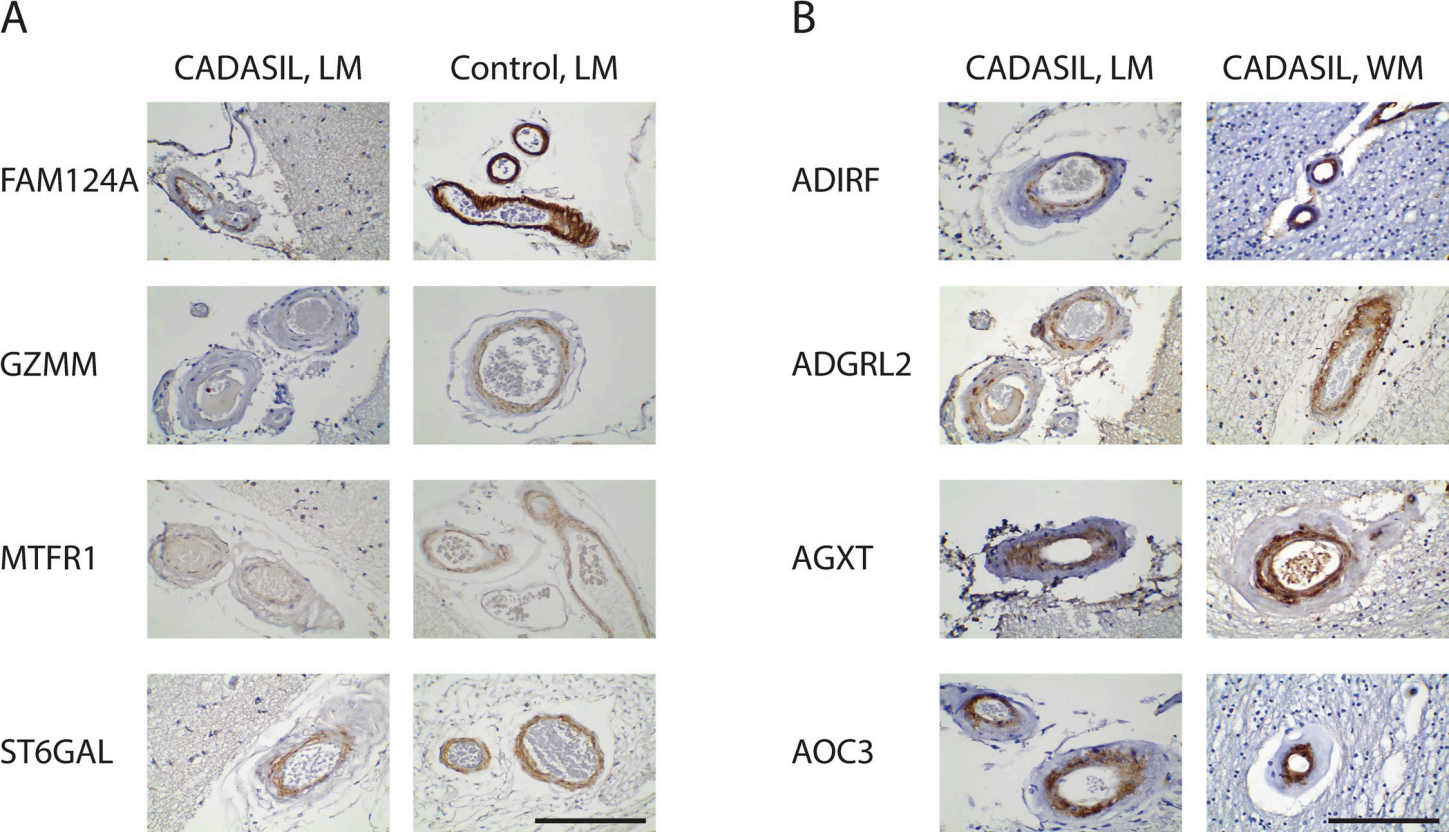
Markers exhibiting differential levels of staining between CADASIL and control arteries or between leptomeninges and white matter. Images from Figs 2 and 3 are placed side by side for easier comparison. A) Immunohistochemical examination of markers (labels to the left) which had decreased staining in CADASIL relative to controls. Representative leptomeningeal arteries (LM) are shown. B) Immunohistochemical comparison of markers (labeled on the left) in leptomeningeal arteries and white matter arteries (WM) in representative CADASIL brain samples. A select group of markers showed moderate white matter enrichment pattern in CADASIL vessels. Scale bar shows 100 microns.

We assessed the level of marker expression by assigning scores of intensity in a blinded fashion. We also assessed the intensity of expression by rank ordering CADASIL and control brains. The expression of markers in control was similar to that of CADASIL with the exception of FAM124A, GZMM, MTFR1, and ST6GAL, which had decreased staining in CADASIL (Fig 4A). None of the scores demonstrated clear-cut increases in CADASIL vs control brain in level of staining.

Although not a criterion for scoring similarity to NOTCH3 staining, we also compared gray matter and white matter artery intensity for 16 SMC markers. There were no clear-cut CADASIL-related differences in gray vs white matter staining intensity. However, the markers ADIRF, AGXT, AOC3, and ADGRL2 showed moderate white matter enrichment pattern in their staining (Fig 4B).

**Fig 5:**
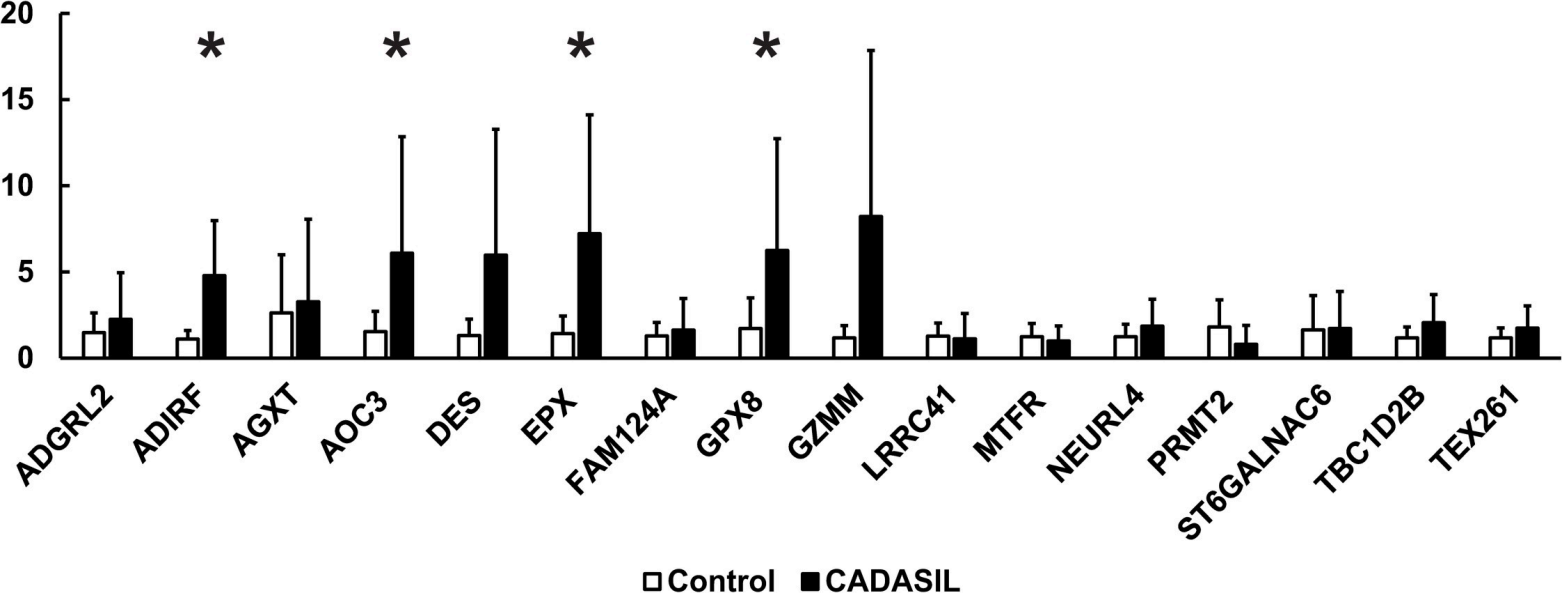
Quantification of mRNA of 16 markers in CADASIL and control brain. RNA from frozen frontal lobe of CADASIL and control brains were analyzed by qRT-PCR; expression values were normalized to 18S RNA and then controls were set to 1 (n = 8 per group; 37% female; average age at death was 62 years for controls and 64 years for CADASIL). X-axis show fold changes relative to controls. Significant differences with a p<0.05 are denoted by asterisks.

To address if there were RNA level changes, we analyzed expression of transcripts corresponding to each of the 16 SMC factors by q-RT-PCR. Of 16 genes tested, the levels of four (ADIRF, AOC3, EPX, and GPX8) were elevated in CADASIL brains compared to controls (Fig 5). The disparity between levels of RNA and protein accumulation could be explained by alterations in protein turnover in the disease. Because CADASIL features protein accumulation in the vascular media in the presence of cell degeneration, it is conceivable that aggregates of proteins could form; these aggregates could last for long periods, long after the decrease in RNA expression. Conversely, some proteins are apparently degraded or not translated well, though RNA is upregulated in CADASIL.

To summarize, the non-canonical 16 SMC protein markers examined in CADASIL tissue exhibited diverse regional patterns of expression and disease-related patterns; however, none of the 16 was found to co-concentrate with pathological NOTCH3 post-translational products in CADASIL.

### Select non-SMC markers concentrate in the media of CADASIL

Because none of the SMC markers showed pathological medial concentration in CADASIL, we extended our analysis to markers highlighted in a published Human Protein Atlas screen and subsequent re-analysis which included probes that highlight vessels in brain [25]. Antibodies, 57 in total, were selected based on avidity of vascular binding to brain tissue and availability from suppliers (Table 2).

**Table 2:** Antibodies against proteins used in phase 2 screen for NOTCH3-like vascular markers in CADASIL. Protein targets are listed alongside antibody catalogue numbers as annotated in the Human Protein Atlas or listed in the Developmental Studies Hybridoma Bank. The presence of a NOTCH3 staining pattern is listed (defined as predominantly medial and enriched in CADASIL arteries). *Two antibodies were delisted from the Human Protein Atlas since the initiation of the project.

Protein List (Sigma)	Catalog number	NOTCH3 pattern
ABCC1	HPA002380	No
ARHGAP20	HPA038459	No
BNIP2	HPA026843	No
C19orf60	HPA043414	No
CASC3	HPA024592	No
CAV1	HPA049326	No
CD63	H5C6	Yes
CD163	MS1103R7	No
CLEC11A	HPA042690	No
CLEC19A	HPA042113	No
CLPS	HPA010512	No
COLEC12	HPA047917	No
CORO7	HPA053586	No
CORO7-1	HPA041657	No
CTSH	HPA003524	Yes
DES	HPA018803	No
DNAH9	HPA020639	No
DRP2	HPA002949	No
EHD3	HPA049890	No
ELOVL7	HPA036337	No
EVI5L	HPA043099	No
EZR	CPTC-EZRIN-1	No
GIPC3	HPA061258	No
GTSE1	HPA060544	No
HRC *	HPA004833	No
IFI30	HPA026650	No
KIAA2022	HPA000407	No
LIMS2	HPA058340	No
LMCD1	HPA024059	No
LMTK3	HPA040203	No
LRRC41	HPA051941	No
MGLL	HPA011993	No
MRC1	AMAB90746	No
MSN1	CPTC-MSN-1	No
MSN2	CPTC-MSN-2	No
MYADML2	HPA041856	No
NAB1	HPA002738	No
NINJ1	HPA045063	No
PHF21A	HPA023580	No
PICALM	HPA019061	No
PLA2G15	HPA041727	No
PRKD3	HPA029529	No
PRKRIP1	HPA051146	No
PRX	HPA001868	No
RDH16	HPA038518	No
RHOF *	HPA060057	No
S100A4	CPTC-S100A4-3	No
SLC2A1	HPA031345	No
SLC9A3R2	HPA001672	No
STRA6	HPA040839	No
SYNPO2	HPA030665	No
TAPBP	HPA007066	No
THEG	HPA042578	No
TTC23	HPA040369	No
UBTD1	HPA034825	No
WDR59	HPA041084	No
YIF1A	HPA014840	No

**Fig 6:**
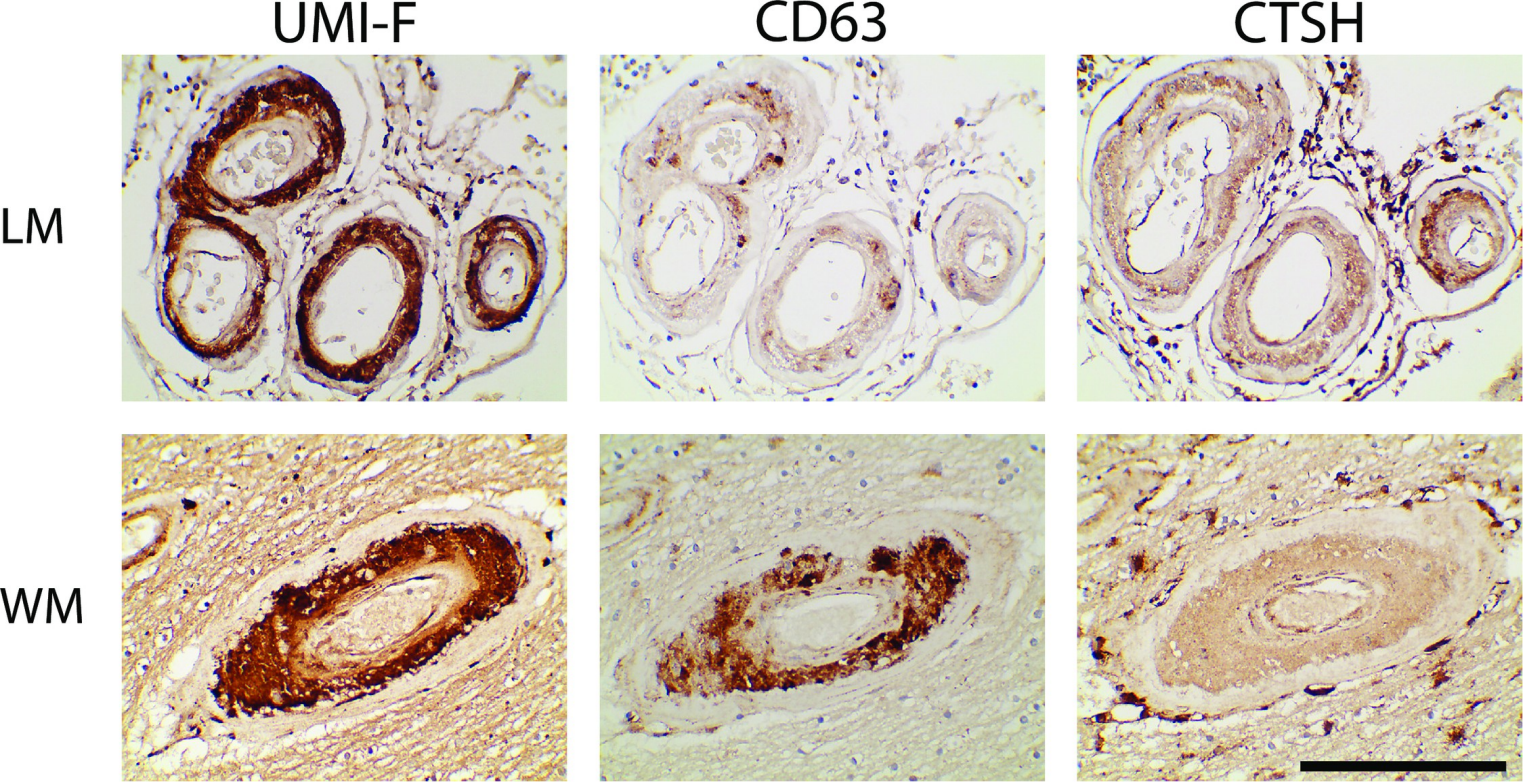
Localization of CD63 and CTSH in CADASIL cerebral arteries. Immunohistochemical localization was performed for the N-terminal fragment (NTF; recognized by UMI-F), CD63 (H5C6), and CTSH on serial sections of CADASIL frontal lobe. Both leptomeningeal (LM) and white matter (WM) arteries showed medial staining which exceeded levels in the adventitia and intima. Scale bar shows 100 microns.

A wide range of expression patterns in CADASIL vessels were noted. Among the non-SMC proteins examined, we found two of the 56 to be concentrated in a pattern that was similar to post-translationally modified NOTCH3. Specifically, CD63 and CTSH (Fig 6) were deposited in the media of leptomeningeal vessels and much more densely expressed in media compared to intima. These markers exhibited increased deposition in CADASIL vs control brain and were also deposited in the same distribution as pathologically processed NOTCH3 in the gray matter and the white matter. In white matter, the markers were strongly expressed in the media of hyalinized vessels, in a region that overlapped with UMI-F reactivity (for the cleavage product that separates the first EGF-repeat from the rest of NOTCH3); the adventitia did not harbor significant amounts of these products.

**Fig 7:**
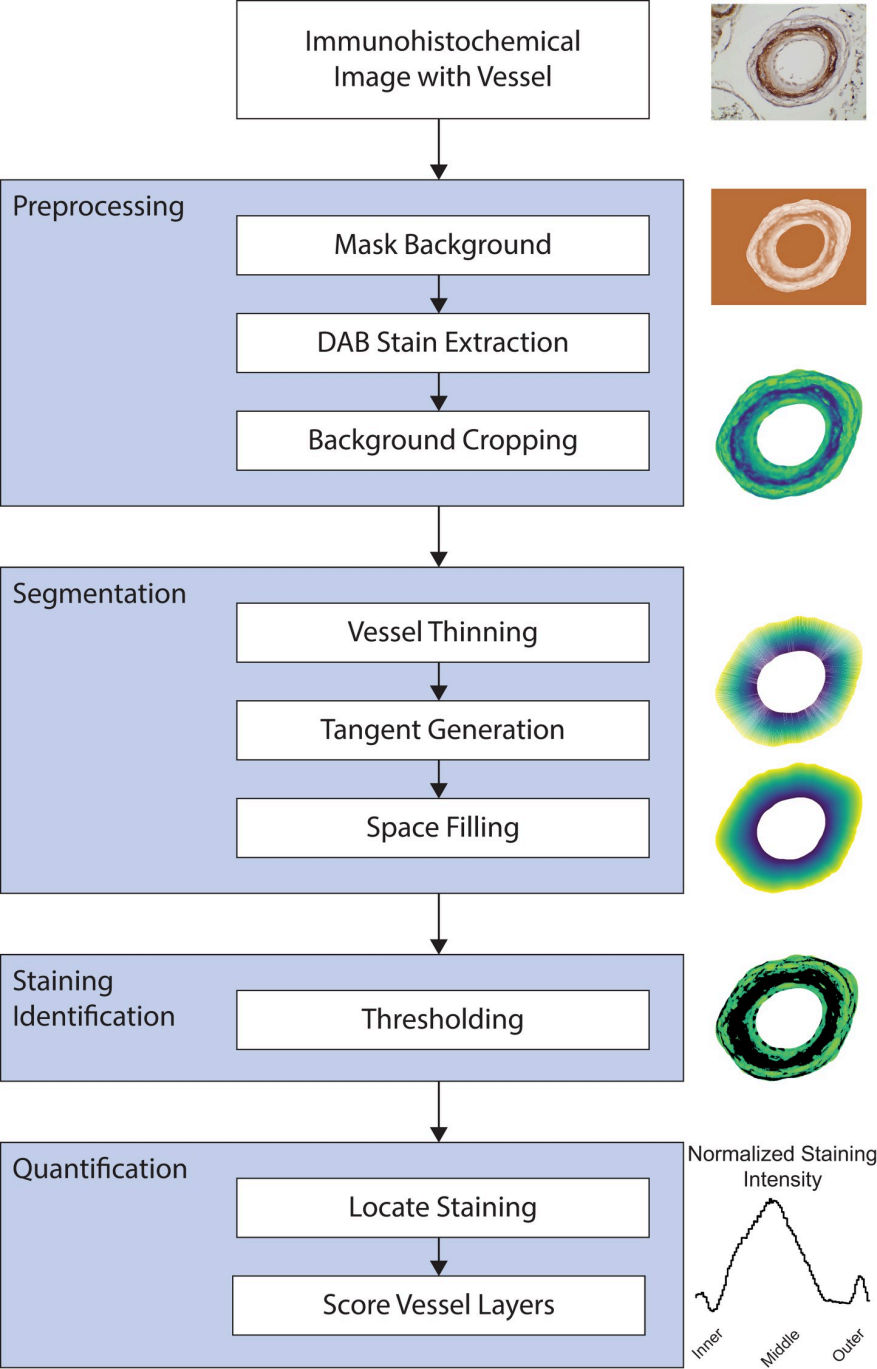
Image analysis workflow depicting VesSeg. A leptomeningeal vessel stained for NOTCH3 was used to demonstrate the process (top panel). The artery was pre-processed using sequential color deconvolution to remove the hematoxylin signal and to focus on the DAB signal (second panel). Masking and then cropping were performed on the processed image to limit analysis to the vessel wall. Spokes were generated for the vessel, resulting in radial segmentation of the wall (third panel). Furthermore, stained regions were thresholded using a custom criterion for processing (fourth vessel). Finally, a histogram depicting the frequency of staining along the vessel wall width was generated (higher frequency corresponds to increased level of staining in that region; last panel).

Immunohistochemical patterns described above were agreed upon by the investigative team that was performed independently and across multiple CADASIL samples (8 or more diseased samples per protein target). Nevertheless, to provide more robust analysis and to quantitatively define protein expression in regions of CADASIL vessels, we developed an image analysis algorithm, called VesSeg, to objectively score distributions of each molecule in intima, media, and adventitia. The approach is summarized in Fig 7. In brief, VesSeg segments the vessel wall into multiple sections along its circumference and width, measures the staining in each section, and groups these sections into the three main layers to allow for comparison.

**Fig 8:**
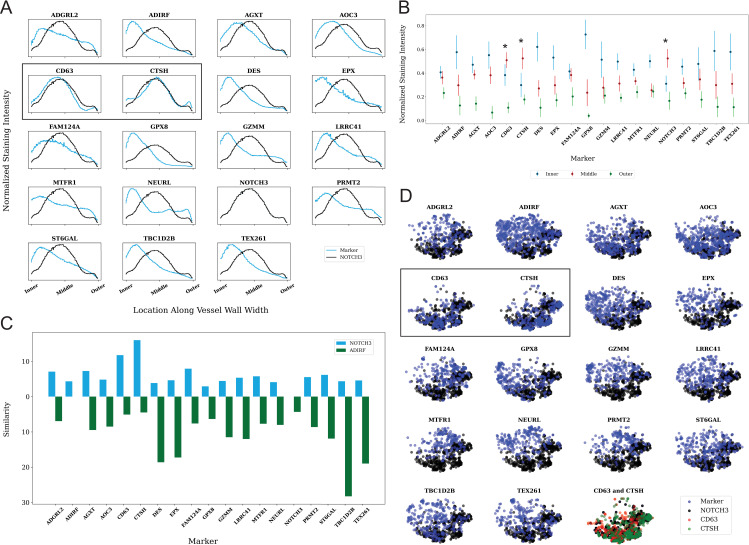
Quantitative analysis of marker deposition in CADASIL arteries using VesSeg. A) Mean staining distribution patterns plotted for each marker. The wall of each vessel was first split into 100 circumferential bands, and then the average staining for each region is calculated. Blue represents the marker distribution, while black indicates the reference NOTCH3 distribution. B) The staining scores for the intimal, medial, and adventitial layers of the blood vessel wall plotted by antibody. After pre-processing and segmentation, signal density maps were divided into three zones corresponding to adventitia, media, and intima. Then, the average pixel value in each layer was calculated and then normalized by subtracting the average value for the entire vessel from it. The normalized score for each layer was plotted along with standard deviation of all the vessels analyzed per marker. Red arrows show proteins which have increased expression in the media of arteries. Significant differences between the tunica intima and media staining is indicated by an asterisk; p < 0.05. C) Similarity scores between each marker and the reference NOTCH3 and ADIRF distributions. The score was calculated as the inverse Jensen-Shannon distance. D) t-SNE plots of vessel wall feature representations comparing each antibody (in black) to NOTCH3 (in blue). We used 100-dimensional vectors corresponding to the staining distribution of tangential sections of the blood vessel analysis to determine how the antibody-stained vessels are represented by our image analysis algorithm using t-distributed stochastic neighbor embedding (t-SNE). Black boxes highlight the t-SNE plot comparisons between markers with expression in the media of vessels (CD63 and CTSH). The final panel is a t-SNE plot comparing the vessel wall feature representations for NOTCH3, CTSH, and CD63 displayed together.

This approach is advantageous because it provides an unbiased approach to assigning regions of highest expression within vessels and uses an objective quantification, which accounts for variations in section reactivity and vessel variability, of regional expression of protein. We assumed that the intima, media, and adventitia were of equal width in all vessels analyzed. Application of VesSeg to the 16 non-canonical SMC markers and to NOTCH3, CD63, and CTSH revealed that only CD63 and CTSH had the most similar mean staining distributions to NOTCH3 (Fig 8A and 8C). Furthermore, NOTCH3, CD63, and CTSH were localized to media, while most of the other markers had staining localized to the intima. This is illustrated in Fig 8B, which shows that these three markers scored highest in the medial region.

We further analyzed the distributions of staining by generating t-SNE plots of all tangential segments of all markers (Fig 8D). The plots of all non-NOTCH3 stains were co-displayed with NOTCH3, demonstrating that overlap with NOTCH3 was most notable for CD63 and CTSH, whereas all other stains generated t-SNE plots that poorly overlapped with NOTCH3. Of note, the t-SNE plots of CD63 and CTSH (Fig 8D) did not overlap, suggesting that differences in expression of each marker within the degenerating media that is not visually apparent.

**Fig 9:**
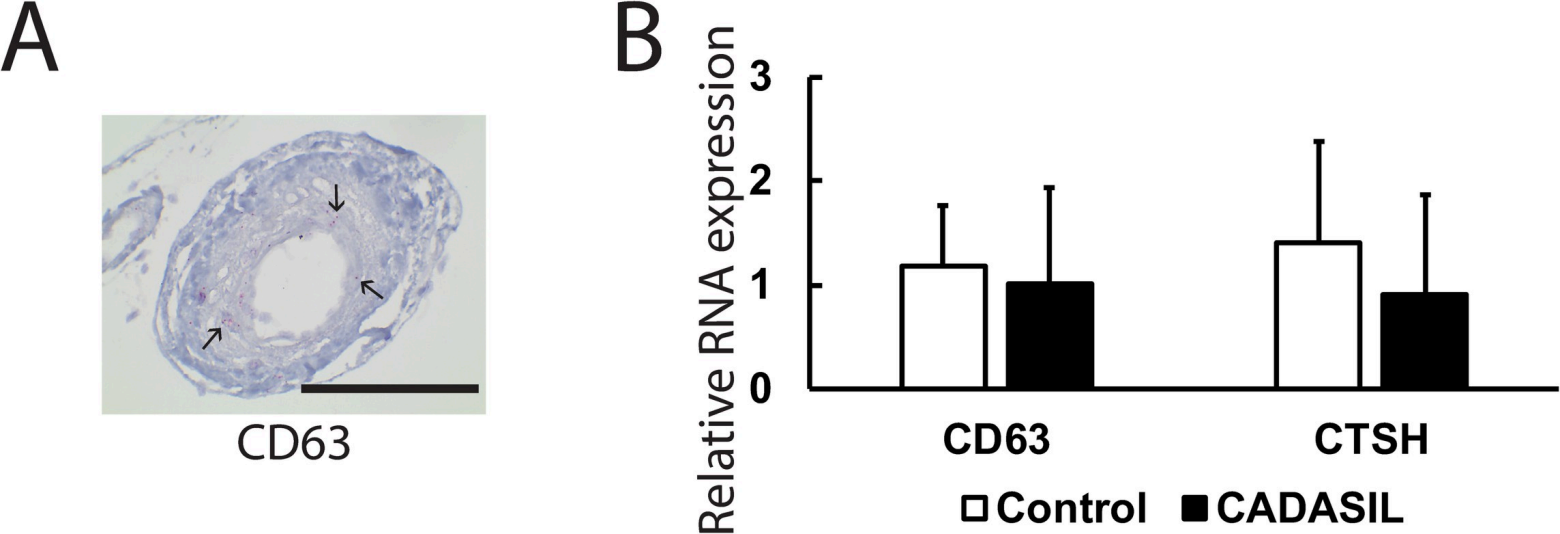
In situ mRNA localization and levels of expression of non-myocyte factors, CD63 and CTSH, in CADASIL arteries. (A) Representative artery which display clear CD63 mRNA signal in the media of leptomeningeal arteries by in situ hybridization. Similar findings were obtained with multiple vessels in several patients. Arrows point to the pink signal of mRNA molecule in vascular smooth muscle layer of arteries. Scale bar shows 100 microns. (B) Quantitative RT-PCR was performed for CD63 and CTSH to assess relative expression of corresponding mRNA in CADASIL and control frontal brain samples.

When RNA expression of CD63 was visualized by in situ hybridization, transcripts for each were observed in degenerating media (Fig 9A), matching the protein expression patterns observed by immunohistochemistry. The overall levels of CD63 and CTSH mRNA were the same in CADASIL and control brain samples (Fig 9B).

**Fig 10:**
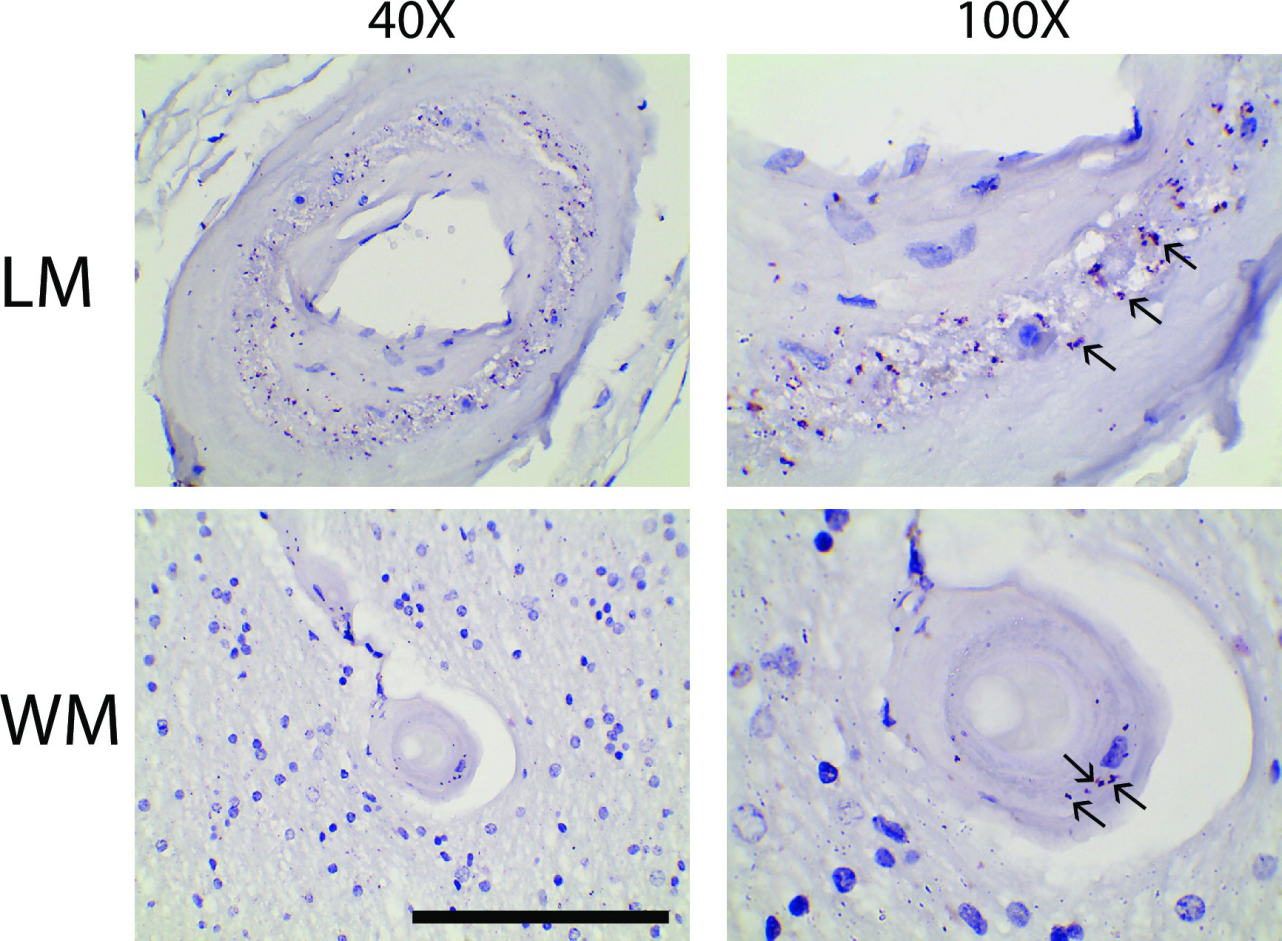
Proximity of CD63 and NOTCH3 fragmentation products in CADASIL. Experiments were performed to assess whether NOTCH3 fragmentation product NTF (recognized by UMI-F antibody) is in close association with CD63 (recognized by H5C6) in tissue. Arrows point to the magenta signal indicating positive PLA signal in the media, in the vicinity of vascular smooth muscle cells. Leptomeningeal (LM) and white matter (WM) arteries are shown. The left side shows 400X images; the right side shows 1000X images of a subregion of the same vessels. Scale bar shows 100 microns in the left panels.

To assess the potential for CD63 to interact with the post-translationally altered products of NOTCH3, we performed proximity ligation assays (PLA) for CD63 and the neo-epitope released by truncation of NOTCH3 between the first and second EGF-like repeats (Fig 10). PLA signal was observed in the arterial media of all patients with CADASIL that were tested. Efforts to co-immunoprecipitate CD63 and NOTCH3 were not successful.

## Discussion

The initiating event in CADASIL is mutation of NOTCH3, which is expected to drive alterations in NOTCH3 protein structure. Consistent with this expectation, structurally altered NOTCH3 products concentrate in the media of cerebral arteries [10, 14, 18]. We now identify additional proteins that co-concentrate with these NOTCH3 products using a candidate-protein approach derived from analysis of the Human Protein Atlas. The results are notable for: 1) the uniform absence of medial concentration of SMC proteins in CADASIL and 2) the identification of two novel media-concentrated CADASIL proteins which are not normally expressed in SMC.

### Location of SMC proteins in CADASIL vessels

In a previous study, we examined the expression patterns in CADASIL of five canonical markers of vascular smooth muscle [22]. We found that none of these markers localized to the same regions as pathological NOTCH3 transformations. The present study significantly extends the number of smooth muscle proteins examined in CADASIL. When we collectively consider the of all proteins in our prior study and the current investigation, 21 of 21 smooth muscle proteins failed to accumulate in regions that coincided with pathological NOTCH3. The exclusion of smooth muscle proteins from the site of major pathology within CADASIL vessels could be accounted for most simply by vascular smooth muscle degeneration and subsequent clearance of SMC proteins from the vessel wall.

In our previous study, we found that five of five canonical SMC proteins were no longer in the media in CADASIL but, instead, were expressed in the intima. In this study, 6 of 16 smooth muscle proteins were concentrated in the intima. In total, 11 of 21 smooth muscle proteins accumulate in the intima, an indication that intimal cells in CADASIL retain many molecular features of mature smooth muscle cells. As noted before, these results demonstrate that the cellular content of the intima in CADASIL is not composed of uniformly dedifferentiated smooth muscle cells that have traditionally been described in peripheral vascular diseases [28–30], since SMC markers are expected to be lost in de-differentiated cells.

### Non-SMC proteins in CADASIL vessels

A principal new finding of our immunohistochemical screen is that two non-SMC proteins concentrate in CADASIL vessels in a pattern that coincides with pathological forms of NOTCH3. Although we do not have functional data on how these proteins are related to NOTCH3, we show that at least one of them (CD63) is in close enough proximity in arteries to bind to truncated NOTCH3. The possibility that CD63 builds up as a response to NOTCH3 or participates in the truncation of NOTCH3 will require additional experimentation to decipher. Another possibility is that CD63, which is a key component of exosomes [31, 32], accumulates in vessels as due to extracellular vesicle deposition.

Further work is also required to understand the significance of CTSH in the vessel wall of CADASIL. As an extracellular protease, a candidate role for CTSH in the context of CADASIL is that it may participate in clearance of proteins or alternatively the post-translational processing of proteins that build up in the vessel wall. It is of interest that mutations in a related cathepsin gene, CTSA, has been implicated in an independent inherited SVD, Cathepsin A-related arteriopathy with strokes and leukoencephalopathy (CARASAL) [33].

In vitro studies that measure the cleavage of recombinant fragments of NOTCH3 have not demonstrated that CD63 or CTSH regulates the cleavage of NOTCH3; improved models of CADASIL that more comprehensively phenocopy the pathological changes of the human disorder will be needed to address this possibility. Future investigations could also query whether mutations in CD63 or CTSH are linked to human cerebral small vessel disease outside of CADASIL.

**Fig 11:**
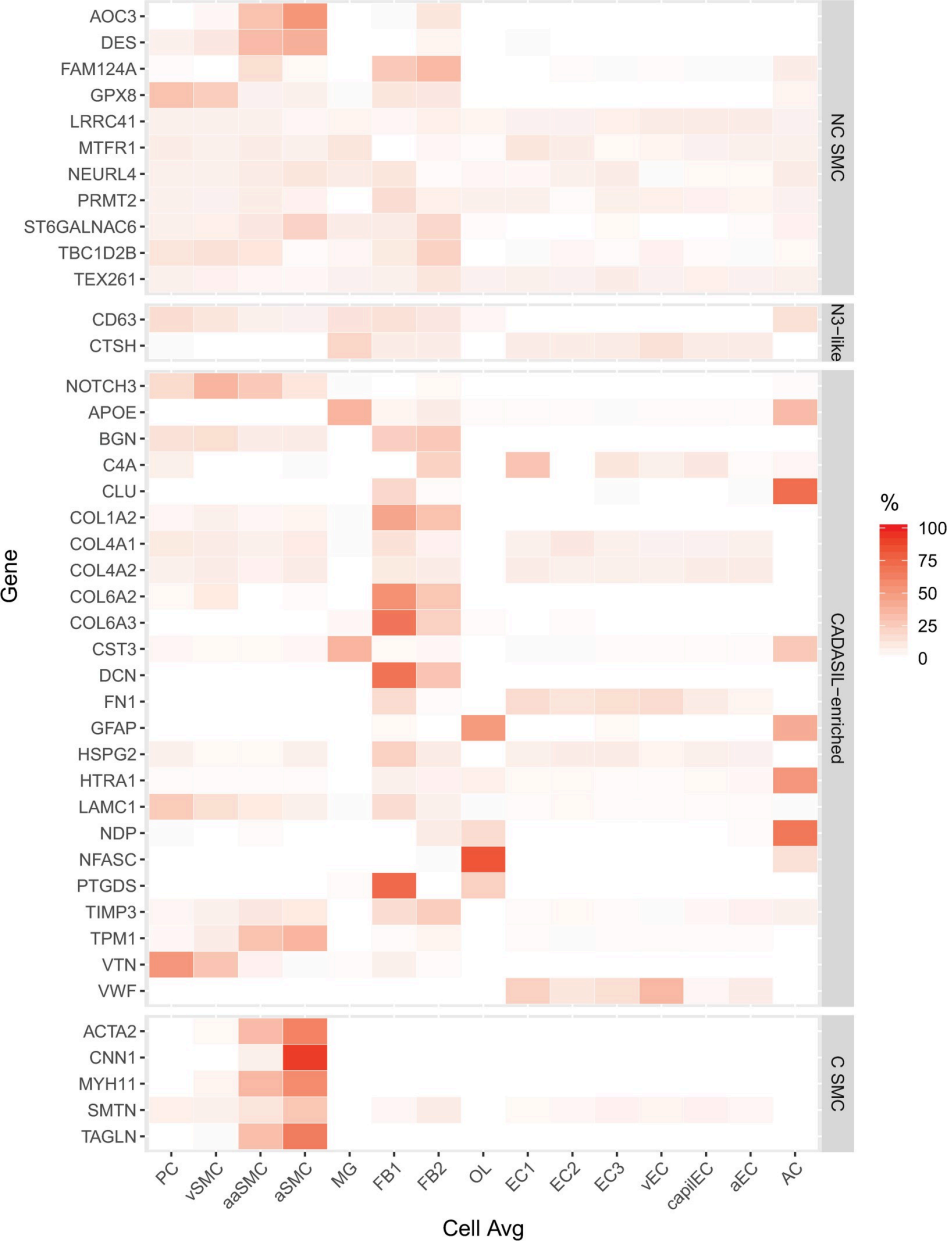
Cellular origin of markers localized in CADASIL vessels. Cell transcriptomic analysis of proteins that accumulate in CADASIL media and of SMC markers. Data was obtained from publicly accessible sets described in [34], in which mouse vessel single cell transcriptomic analysis was performed. All values shown in the heatmap represent fraction of expression normalized to the total number of counts reported across all cell types examined. Marker names are arrayed on the y-axis and cell types along the x-axis. The markers were grouped to cluster non-canonical SMC markers (NC SMC), newly identified NOTCH3 distribution proteins (N3-like; CD63 and CTSH) with NOTCH3, previously identified molecules that were identified in CADASIL vessels [21] (CADASIL-enriched), and canonical SMC markers (C SMC).

Both of the newly described CADASIL media-concentrated proteins in this work do not appear to be markers of SMC. To further investigate cellular origin of CD63 and CTSH, we queried public single cell RNA-seq data from mouse brain vessels [34]. Normalized distribution of message encoding these two molecules shows broad distributions across cell types of the brain (Fig 11; N3-like). In contrast, the distributions of message for canonical and non-canonical SMC markers were significantly more highly enriched in arterial smooth muscle cells (Fig 11; C SMC and NC SMC).

To compare CD63 and CTSH cellular expression to the distributions of messages encoding previously described factors found in CADASIL vessels, we display single cell RNAseq data in Fig 11. These distribution patterns were also broadly distributed outside of SMC (Fig 11; CADASIL enriched) relative to SMC, with expression patterns that are easily differentiated from canonical SMC markers (Fig 11; C SMC). Of note, a majority of the molecules found in CADASIL pathological media have significant expression in fibroblast cells, a cell type that does not express canonical SMC proteins. These data suggest that proteins identified to date that accumulate in diseased media in CADASIL are likely to be derived from cells of non-SMC origin.

The finding that proteins that co-concentrate in the media with pathological NOTCH3 have origins from non-smooth muscle cells suggests that understanding pathological changes that occur in non-SMC cell types, particularly fibroblasts, may impact our evolving understanding of cerebral small vessel disease.

Finally, we highlight the availability of VesSeg, a newly constructed algorithm for quantifying the distribution of staining in pathological vessels. VesSeg was designed specifically for quantifying the layer of vessel where a marker was most abundantly expressed. This approach has face validity as it reflected the visual scoring of staining results and was able to categorize NOTCH3, CD63, and CTSH patterns as similar. At the same time, it confirmed that 16 additional markers were not similar to NOTCH3. Because it is an objective algorithm that provides quantification, it has the potential to be used for larger scale projects and in the disease investigations. Furthermore, based on t-SNE analysis, higher order information is included in this algorithm that may, in future work, point to more complex patterns of smooth muscle protein expression in CADASIL vessels. Such new approaches may be helpful to determine the origin and significance of proteins, like CD63 and CTSH, that appear alongside NOTCH3 in CADASIL.

## Supporting information

S1 File Contains all the supporting text and S1 and S2 Figs. (PDF)
