# Supplementary material for: Concentration of non-myocyte proteins in arterial media of cerebral autosomal dominant arteriopathy with subcortical infarcts and leukoencephalopathy
Source: PLoS One. 2023 Feb 8;18(2):e0281094. doi: 10.1371/journal.pone.0281094 (PMC9907840; doi:10.1371/journal.pone.0281094)
Supplement: S1 File — (PDF) [file pone.0281094.s001.pdf]

<u>Supplemental Material</u>: Additional description of VesSeg algorithm

The FIJI program suite was used to initially preprocess the images [1] [https://imagej.net/software/fiji/] . First, the regions inside and outside the blood vessel wall were masked, converting these regions to a constant value. This also included extraneous tissue adjacent to the wall. Deterioration of the tunica adventitia was prevalent, making it difficult to delineate the wall boundaries properly–this may have affected analyzing the vessel. Next, a color deconvolution algorithm was used to separate the stains present in the image [26]. This step was performed because only the DAB stain–the stain representative of the protein of interest–is relevant. Note the filtered stains are now represented as a grayscale image. This is the end of the manual steps involved in the image analysis.

First, the value of the masked background needed to be determined for future processing steps. This was assigned the most common pixel value in the image under the assumption that the background makes up the majority of the image, which was true for all images analyzed in this study.

Next, the masked background external to the blood vessel wall was cropped automatically with the following procedure:

1. Start along the rightmost edge of the image.
2. Select the most external pixels along that edge (i.e. in this case, choose the rightmost column of pixels).
3. If all of the pixels' values are the same, then remove the row.
4. Repeat Steps 1-3 until the condition in Step 3 is not true.
5. Repeat Steps 1-4 for the other 3 edges.

Note this process does not and is not meant to remove all of the external backgrounds.

At this point, the algorithm does not know what parts of the image correspond to the vessel wall or the masked background. More specifically, the regions corresponding to the lumen of the blood vessel, the blood vessel wall, and the space surrounding the blood vessel need to be differentiated for future processing steps. The former and latter will now be referred to as the inner and outer regions, respectively. Note that these regions have already been masked as part of the background. Since the background has a constant value, we utilized OpenCV's implementation of the FloodFill algorithm (the floodfill method) to determine these regions [opencv.org].

The flooding algorithm “fills” an area of interest with a matching attribute. For example, in this case, the area of interest is a region with the same pixel value and is separated from another similar region. With a seed pixel within the region of interest, this algorithm can determine all pixels directly and indirectly connected to the starting pixel with the same value. The outer region was resolved by using the four corners of the image as starting points for the flooding algorithm. Next, the inner region was demarcated with the following procedure:

1. Choose all pixels in the image not equivalent to the background value, leaving the area containing the vessel wall.
2. Combine the previous area determined with the outer region.
3. Utilize the combined area to mask out the respective regions (outer wall and blood vessel wall), yielding a region representative of the inner region. Note that this region may include pixels in the blood vessel wall as it is not assumed that the wall does not include pixels equivalent to the background value.
4. To alleviate this problem, the flooding algorithm was used to determine the area corresponding to the inner region by using the centroid of the area determined in Step 3 as the starting point.

The vessel wall region can now be determined by masking out the outer and inner regions.

The next step in VesSeg is to segment the vessel. Circumferential segmentation of the vessel wall was accomplished by generating "spokes" perpendicular to the inner and outer boundaries to quantify the location of pixels within the wall relative to them. The spoke generation algorithm involves estimating a spoke at each inner boundary. Note that the definition of point in this context is discrete and equivalent to a pixel. Below is a detailed description of the process (Supplementary Figure 2A):

1. An offset was first determined to estimate the spoke. Here, the offset parameter is equal to 4% of the number of points along the inner boundary. This value was determined through parameter testing across a variety of sample vessels.
2. First, a parallel line needs to be estimated at the point of interest. This was accomplished by selecting two points, both X points away from the point of interest in the right and left directions, where X is the offset parameter. For example, if the offset was 10, then the 10th points to the left and to the right of the point of interest. Denote these points as the *offset endpoints*.
3. Then, the angle of the offset endpoints with respect to the point of interest was determined by transforming the endpoints relative to the point of interest and converting them to polar coordinates. To account for the nature of the transformations, the angle was modified accordingly.
   - To be specific, if the left endpoint had a lower x-coordinate than the right endpoint, then π was added or subtracted to the angle if it was negative or positive, respectively.
4. The angle of the spoke was calculated by transforming the parallel angle π/2 clockwise.

5. The last step of the algorithm is to find the point along the outermost boundary that is closest to this angle with respect to the point of interest.
   - To determine which point was the closest in terms of angles, the outer boundary points were transformed with respect to the point of interest and converted to polar coordinates.
6. The spoke has now been determined. Now, apply Steps 1-5 to the rest of the inner boundary points.

Assuming the spoke is perpendicular to the boundaries of the vessel wall, the location of the points with respect to the vessel width can be determined. For example, all midpoints of the spokes must also lie along the midline of the vessel wall. We can assign each point a value representing this information through the following method:

1. Assume there are N points in the spoke.
2. Index the points in the spoke starting from the inner boundary starting from 0. To be more precise, assign a value of 0 to the inner boundary point, 1 to the next point, and continue this process until the last point, which should lie on the outer boundary.
3. Now, apply the following formula to each of the points' indices: (index + 1) / N. With this, each point has been assigned a score that designates its relative location along the vessel wall width.

Furthermore, in the methods that estimate spokes by finding perpendicular lines from a surface, the location of each point along the spokes relative to the vessel wall circumference can also be determined by indexing the points composing the original surface. This information is

useful in terms of locating a point with respect to a vessel's circumference. From now on, we will denote values corresponding to a pixel's location relative to the vessel wall width and circumference as its distance and angle, respectively (e.g., similar to the definition of a polar coordinate).

Now, an area of interest can be selected by indexing with these values. For example, if points near the middle of the vessel wall are desired, this can be accomplished by selecting points with values between 0.4 and 0.6. However, the spokes constructed thus far do not include all the points in the vessel wall. The following process assigns values to the remaining points by utilizing information from the spokes (Supplementary Figure 2B):

1. Select all points in the vessel wall that have not been assigned a value. Denote these points as *empty*.
2. Iterate through each empty point and apply the following steps:
    a) Select all points that are 1 pixel away from the empty point (in all directions and diagonally). Denote these points as a *neighborhood*.
    b) If there are more than 2 nonzero elements (non-empty points) in the neighborhood, then set the empty point to the mean of the nonzero elements.
3. Repeat steps 1-2 until there are no empty points remaining.

This process is utilized to define both location and angle of the empty points. In rare cases, not all empty points can be filled, and the image is disregarded.

Finally, a custom thresholding criterion was used to analyze only the stained regions within the vessel wall:

1. Calculate a binary threshold with the Minimum Error Thresholding (MET) method [2]
2. If this threshold is in the upper 25% of pixel intensities, then assume the image is not stained. (The original assumption is that the distribution is unimodal, which either means the vessel has a uniform or no staining. In this study, no staining was assumed.)
3. If not, then apply k-means clustering with a k=3 to the vessel wall. The clusters were assumed to correspond to darkly stained, lightly stained, and non-stained regions in decreasing average pixel intensity.

The spatial information from the segmentation algorithm can be used to determine where the stained regions occur in the vessel wall. Specifically, a histogram was constructed using the location relative to the vessel wall width of all pixels found in the stained regions, with the number of bins set to 100. One way to conceptualize this is to imagine the vessel wall was segmented into 100 evenly-spaced bands, and the number of stained pixels in each band was counted. To account for differences between similarly stained regions, darker stained pixels were overrepresented in the histogram according to their inverse pixel intensity (original pixel intensity subtracted from 255 because stained regions are originally assigned lower values). For example, if two pixels had starting intensities of 150 and 100, 105 and 155 of each were counted towards the histogram, thus weighting the darker pixel more. Then, the histogram was normalized by dividing each value by the number of total pixels in each band by the sum of the entire histogram. The result is a normalized probability distribution with a sum of 1. The staining distribution for a protein was calculated by summing the probability distribution for each vessel followed by normalization.

REFERENCES

1. Schneider CA, Rasband WS, Eliceiri KW. NIH Image to ImageJ: 25 years of image analysis. Nat Methods. 2012;9(7):671-5. doi: 10.1038/nmeth.2089. PubMed PMID: 22930834; PubMed Central PMCID: PMCPMC5554542.

2. Kittler J, Illingworth J. Minimum error thresholding. Pattern Recognition. 1986;19(1):41-7. doi: https://doi.org/10.1016/0031-3203(86)90030-0.

Supplemental Figure 1 (S1). Visualization of quantification and segmentation terms defined. Masking of image background is represented by gray regions. Definition of areas, bands, and patches within vessel wall are shown.

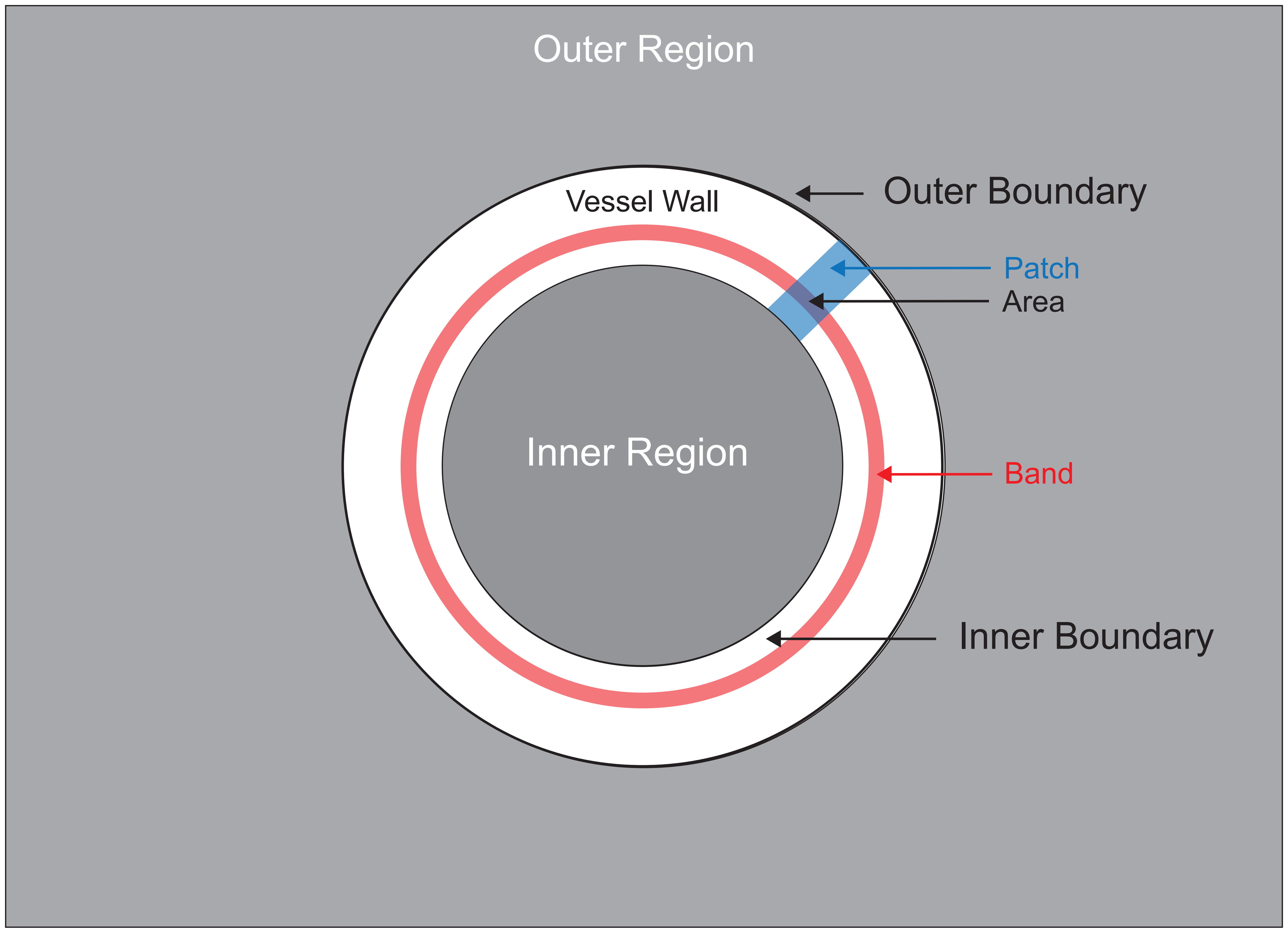
Outer Region
Vessel Wall
Outer Boundary
Patch
Area
Inner Region
Band
Inner Boundary

Fig S1

Supplemental Figure 2 (S2). Depiction of algorithms used in VesSeg. A) Flowchart of spoke generation method used. First, the thinning algorithm is applied to the vessel wall and pruned, resulting in a skeleton (indicated by the gray dashed line). Next, seed points are chosen along with the skeleton for spoke generation. The direction of the spoke is determined by finding the perpendicular angle of points offset to the seed point at a specified distance. Finally, points along the boundaries closest to this direction are selected, completing the spoke. B) Flowchart of the space-filling algorithm applied to a 5x5 area. Each individual cell represents a pixel, and the bolded 3x3 region represents the current window of the empty point being filled with the mean of its neighboring non-empty points. Blue represents pixels that are part of spokes (and thus whose values are already determined); green presents pixels that were previously empty, and red represents empty pixels.

A

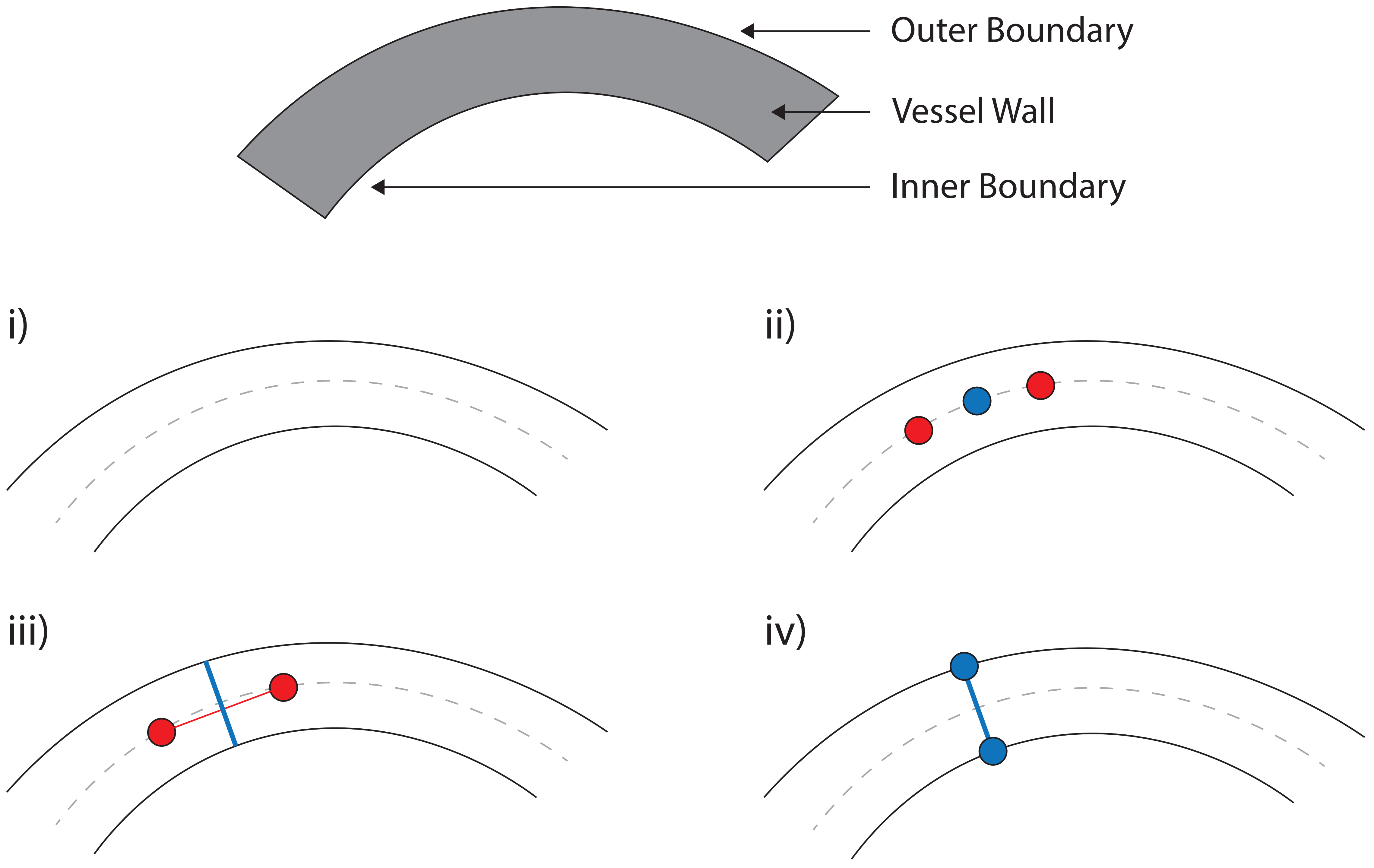

B

1

0.68	0.67	0.67	0.66	0.66
0.66	0.65	0	0	0.64
0.64	0.63	0	0	0.62
0.62	0.61	0	0	0.60
0.60	0.59	0.59	0	0.58

→

0.68	0.67	0.67	0.66	0.66
0.66	0.65	0.66	0	0.64
0.64	0.63	0	0	0.62
0.62	0.61	0	0	0.60
0.60	0.59	0.59	0	0.58

2

0.68	0.67	0.67	0.66	0.66
0.66	0.65	0.66	0	0.64
0.64	0.63	0	0	0.62
0.62	0.61	0	0	0.60
0.60	0.59	0.59	0	0.58

→

0.68	0.67	0.67	0.66	0.66
0.66	0.65	0.66	0.66	0.64
0.64	0.63	0	0	0.62
0.62	0.61	0	0	0.60
0.60	0.59	0.59	0	0.58

3

0.68	0.67	0.67	0.66	0.66
0.66	0.65	0.66	0.66	0.64
0.64	0.63	0	0	0.62
0.62	0.61	0	0	0.60
0.60	0.59	0.59	0	0.58

→

0.68	0.67	0.67	0.66	0.66
0.66	0.65	0.66	0.66	0.64
0.64	0.63	0.64	0	0.62
0.62	0.61	0	0	0.60
0.60	0.59	0.59	0	0.58

Fig S2
